# Infinitely long branches and an informal test of common ancestry

**DOI:** 10.1186/s13062-016-0120-y

**Published:** 2016-04-07

**Authors:** Leonardo de Oliveira Martins, David Posada

**Affiliations:** Department of Biochemistry, Genetics and Immunology, University of Vigo, Campus Universitario, Vigo, 36310 Spain; Department of Materials, Imperial College London, Prince Consort Rd, South Kensington, London, SW7 2AZ UK

**Keywords:** Universal common ancestry, Alignment uncertainty, Star tree, Model selection, Selection bias

## Abstract

**Background:**

The evidence for universal common ancestry (UCA) is vast and persuasive. A phylogenetic test has been proposed for quantifying its odds against independently originated sequences based on the comparison between one versus several trees. This test was successfully applied to a well-supported homologous sequence alignment, which was however criticized on the basis of simulations showing that alignments without any phylogenetic structure could mislead its conclusions.

**Results:**

Here we present a simplified version of this same counterexample, which can be interpreted as a tree with arbitrarily long branches, and where the UCA test fails again. We also present another case whereby any sufficiently similar alignment will favour UCA irrespective of the true independent origins for the sequences. Finally, we present a class of frequentist tests that perform better than the purportedly formal UCA test.

**Conclusion:**

Despite claims to the contrary, we show that the counterexamples successfully detected a drawback of the original UCA test, of relying on sequence similarity. In light of our own simulations, we therefore conclude that the UCA test as originally proposed should not be trusted unless convergence has already been ruled out a priori.

**Reviewers:**

This article was reviewed by Professor Eugene Koonin, Dr. Yuri I. Wolf and Professor William Martin.

**Electronic supplementary material:**

The online version of this article (doi:10.1186/s13062-016-0120-y) contains supplementary material, which is available to authorized users.

## Background

Douglas Theobald [1] proposed a quantitative test (from now on, the UCA test) to distinguish common ancestry (CA) from independent origins (IO) of a set of aligned sequences, by modelling CA as a single tree connecting all sequences against two or more trees representing IO. To proceed with the actual calculations, nonetheless, the same single alignment represented both hypotheses – which did not matter for the specific, highly curated data set he analysed. However, we and others have raised concerns that such a test would mistakenly infer homology (common ancestry) whenever the sequences are sufficiently similar [2–6], rendering it suspicious for alignments of arbitrary quality. In particular Koonin and Wolf [3] (K&W) presented a counterexample where alignment columns did not follow any phylogenetic structure and were simply sampled from a pool of amino acid frequencies. This simulation model, called “profile” model in [7], was enough to skew the original UCA test into preferring UCA. Theobald defended his test replying that his method would work as advertised once extended to include the true generating model of the simulated counterexamples, and also concluded that the criticisms did not apply for his “very high confidence alignment” [7].

We have already shown that the UCA test fails even for sequences simulated exactly under the described models of CA and IO, due to the obligatory alignment optimization step [2]. We also commented on the arbitrariness of resorting to sequence similarity justifications, since all examples where the UCA test favoured IO had very low pairwise similarity [2], not to mention that such a requirement would imply in a unacceptable selection bias [6].

Here we explain why the K&W model was a legitimate simulation of IO, showing that the UCA test fails even for a simplified version of this model where the true substitution model is amongst the tested ones. We also simulated IO alignments that satisfy the elusive constraints of quality/similarity imposed in [7] and conclude that UCA will be favoured whenever the sequences are not clearly unrelated. Furthermore, we discuss about the lack of mathematical justification for comparing likelihoods between different alignments, and illustrate it with a simulation showing that the UCA test would fail even if we compare sequences aligned independently. Finally, we introduce a class of frequentist tests that supersede the original UCA test.

## Koonin and Wolf’s profile model

K&W simulated alignments where the amino acid states for each column came from a given distribution of equilibrium frequencies – that is, the state for each taxon at the *i*-th site was sampled from a discrete (categorical) distribution $\boldsymbol {\pi }^{[i]}=\left (\pi _{A}^{[i]},\pi _{R}^{[i]}, \hdots, \pi _{V}^{[i]}\right)$. In this case the original UCA test failed, since the log-likelihood of the whole simulated data set was always superior than the sum of the log-likelihoods of arbitrarily split sequences. Theobald [7] correctly pointed out that K&W’s sequences might “have evolved according to a star tree with equal branch lengths” under a MAX-Poisson evolutionary model [8], but mistakenly assumed that this was equivalent to an UCA scenario. The star tree from K&W model has all branch lengths equal to infinity, as we will see, which means IO and not UCA. There is a key distinction between finite and arbitrarily large branch lengths, which is what ultimately discriminates UCA and IO under the original modelling (see Additional file 1: Appendix – or ([2] Supplementary Text)).

We can verify that K&W simulation corresponds to a star tree under an IO scenario by using, for instance, Equation 1 of [9], which describes a similar model:

(1) $$ P(b\mid a, t)= e^{-t} \delta_{(a=b)}+(1-e^{-t})\pi_{b}  $$

where *a* and *b* are respectively the initial and final amino acid states along a phylogenetic branch of length *t*, *δ*_*χ*_ is the indicator function^1^, and *π*_*x*_ is the equilibrium frequency of state *x*∈(*A*,*R*,…,*V*). A star tree has only one internal node, connected directly to all extant sequences.

As we can see, the probability of observing state *b* under such a star tree is influenced by the initial state *a* until $e^{-t}\rightarrow 0$, which happens at $t=\infty $ and therefore representing IO. For any finite branch length $t<\infty $ the terminal states will still be correlated to the state at the root, shared among them. It’s easy to imagine that for very short branches, the state at the tips of the star tree should be very similar, since they will mostly be the same as the state at the internal node.

The source of Theobald’s confusion might be that although the instantaneous substitution rate does not depend on the current state of the Markov chain, the probability of change over an arbitrary time interval does [10]. In [11] for example it is shown that even for only two sequences the probability of observing state *a* in both sequences at a particular position is given by $e^{-t}\pi _{a} + {\pi _{a}^{2}}(1-e^{-t})$, while the probability of observing state *a* in one sequence and state *b* in the other at the same column equals *π*_*a*_*π*_*b*_(1−*e*^−*t*^).

Therefore, for small time intervals we should expect all sequences simulated under this star tree to be very similar (reflecting the common ancestry with the sequence at the root), while for longer branches they should diverge from one another until the equilibrium frequencies are reached. Under K&W’s model the probabilities of observing the same state *a* or distinct states *a* and *b* are, respectively, ${\pi _{a}^{2}}$ and *π*_*a*_*π*_*b*_, which are equivalent to the star tree model above only when *e*^−*t*^=0, as we saw before. Therefore, K&W’s model corresponds to a star tree where all branch lengths are infinitely large – that is, the sequences are unrelated to their common ancestor and have IO.

A different question is whether we can reliably estimate all parameters from the K&W simulations. The overall poor fit of MAX-Poisson as described in [8] lead us to conclude that we can’t, due to over-parameterization being especially misleading when the number of sequences is small. In this case there is simply not enough data to reconstruct the true amino acid frequencies. It might be the case that a particular data set can by chance have a corresponding phylogenetic model with finite branch lengths that explains the data equally well. But this is not the same as claiming that the data set came from such a common ancestry model.

In a nutshell, K&W’s simulations are equivalent to a MAX-Poisson model over a star tree, but with infinite branch lengths since each sequence is independent from the others. It is worth noticing that this star tree is equivalent to any other tree, or to no tree at all, due to the vanishing branches. And therefore K&W’s simulated sequences are truly originated independently, *contra* Theobald [7]. To claim otherwise would defeat, by the way, the whole phylogenetic model selection framework developed in [1]: if, for each alignment column, sampling the state (of 2 sequences or more) from a common distribution renders the data related by common ancestry, then the idea that two independent trees can represent IO would be wrong since their root positions might be two such ancestral sequences, whose columns came from an “ancestral soup” of amino acids – as we show in the (Additional file 1: Appendix).

To see it from another perspective, we can imagine any two sequences simulated by K&W as the roots of independent phylogenies. If sampling from a common pool of amino acid frequencies was enough signal for common ancestry, then the IO model in [1] (of at least one infinite branch length) would be wrong, since it does not impose restrictions on the IO evolutionary models at the root. The point is that for any combination of phylogenetic models, the IO assumption as devised in [1] is mathematically equivalent to an infinite branch connecting the nodes (apical or not). A more recent CA test explores explicitly this relation between the ancestral root states of two trees [12].

### Our simplified simulation: a homogeneous Poisson+F

To avoid the confusion with the overly parameterized MAX-Poisson model, we reproduced K&W’s simulations but this time using a homogeneous Poisson model – that is, all columns *i* share the same equilibrium frequencies ***π***^[*i*]^=***π***=(*π*_*A*_,*π*_*R*_,…,*π*_*V*_). We simulated 8 sequences with 1000 sites under a randomly sampled vector of shared amino acid frequencies. More specifically, we used INDELible [13] to simulate 2 quartets with a collapsed internal branch of length zero and all terminal branches with a huge length of 2500 – computationally equivalent to 8 independently originated sequences from a common pool of amino acids.

**Fig. 1:**
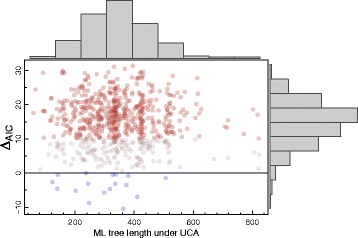
*Δ* *A* *I* *C* values for the simplified version of Koonin & Wolf’s simulations. Positive values for *Δ* *A* *I* *C* favour UCA, and to ease interpretation the simulations favouring IO are displayed as *blue dots*, while those strongly favouring UCA (*Δ* *A* *I* *C*>10) are *red*. Marginal histograms are also shown, and the grey dots represent simulations favouring UCA only slightly

The Akaike information criterion (AIC) model selection analysis was done with ProtTest3 (version from 18/Oct/2010) under a subset of available models, where we included the Poisson model used to generate the data. We were careful to include the true generating model among those tested by the model selection procedure, to be charitable and avoid misspecification issues^2^. To be consistent with K&W we did not optimize the alignment for this analysis, although we know that the test would fail if we aligned them [2]. But while K&W used a set of empirically observed amino acid frequencies to sample from, we simulated these frequencies ***π*** from uniform distributions – each simulated data set had a distinct frequency set, but all sites within a simulation shared the same values. We use *ΔAIC*=*AIC*(*IO*)−*AIC*(*UCA*), such that positive values of *ΔAIC* favour UCA, and a difference in AIC larger than 10 indicates that the model with larger AIC has practically no support when compared to the smaller one [14, 15]. Figure 1 shows that in most (>97 *%*) of our simulations the UCA was wrongly favoured according to the UCA test, despite the large tree lengths making us suspicious about these data. Not only that, 75 *%* of the replicates showed **very strong** support for the wrong hypothesis (that is, considering only those with *ΔAIC*>10).

### A note on pairwise versus phylogenetic comparisons

In his recent reply to K&W, Theobald gave arguments favouring Bayesian model selection over frequentist analysis [7]. But within his overview there seems to be some confusion about the advantages of his method against BLAST-based e-values. One thing are the virtues of Bayesian over frequentist analyses. Another, completely different issue, is the superiority of phylogenetic against pairwise sequence comparisons. Theobald’s own solution to “estimate an upper bound for the effect of alignment bias” was a (frequentist) random permutation of the sequences [7], which weakens his discourse against frequentist methodologies. And as we will see below this advice is incorrect anyway since we cannot compare likelihoods between different data.

Theobald compared a Bayesian phylogenetic model selection with a pairwise null hypothesis framework, praising the former over the latter [7]. But we would still prefer a *frequentist* phylogenetic model over a *pairwise* Bayesian one. That is, we might have a Bayesian model for pairwise homology detection [16] or a frequentist phylogenetic model selection, like for instance the one we present below. Likewise, we could devise a classic hypothesis testing where H0 and H1 are as described in the Additional file 1: Appendix, using a branch length fixed at infinity against the alternative hypothesis with the length free to vary – we do not need to impose the same replacement matrix or other parameters across branches. In all cases the phylogenetic approach should be preferred over pairwise comparisons, because we expect that the effect of using the whole data at once should be more relevant than the statistical framework we choose.

## Data sets conditioned on similarity

Theobald also suggested that his test works without corrections only for “unbiased” alignments [7], which we interpret as being those with low uncertainty and/or composed of very similar sequences – this alignment quality requirement of the test was never formally described. He mentioned “eliminating any potential alignment bias”, where ‘bias’ refers to “artifactually induce[d] similarities between unrelated sequences” ([7] page 14). But to solve the UCA vs IO question we cannot restrict eligible data sets based on similarity, as by doing so we would be introducing an ascertainment bias towards alignments where UCA is more likely than for less similar ones. And notice that this is not to assume that similarity implies in homology, but it is a simple recognition that there is a correlation between similarity and homology that cannot be neglected by excluding the sequences capable of refuting any of the hypotheses tested [6]. And we could even speculate that once we remove the “alignment bias and uncertainty” what we are left with are columns that share a common ancestor *a fortiori*.

It could be finally argued that the UCA test was designed instead only to distinguish UCA and IO from alignments that appear to favour UCA – that is, given similar-looking (or with elusively defined good quality) sequences, the test could detect independently originated sequences. However, Theobald himself did not seem to consider this idea in his examples where the UCA test favoured IO, as the experiment described in the last paragraph of page 221 and in the supplementary subsection 3.1 of [1]. In these experiments, alignment columns for a clade were randomly shuffled, resulting in very low pairwise similarity [2].

Theobald claimed that his test worked “without assuming that sequence similarity indicates a genealogical relationship” [1], so we were interested in checking whether his test can indeed distinguish similar sequences with IO from similar sequences with an UCA. Indeed, it is hard to devise a simulation scenario where sequences generated under IO are very similar to each other, or are free from “alignment bias”, and we have shown that all previous attempts failed at showing the correctness of the UCA test [2]. We have argued that even summary statistics contain information about the likeliness of UCA, and therefore any common ancestry test should take this information into account [6]. Nonetheless, it might be ultimately claimed that only bias-free alignments could invalidate the UCA test. Maybe a simulation where independent sequences should converge to a similar protein structure or to a limited set of structures might fit the demands, but we do not know how to properly implement such a model at this point.

The closest approximation we could devise was to repeat the IO and UCA simulations as in [2], but now selecting the columns such that the average identity was above a given threshold. We must recall that this is not a proper simulation of highly similar IO sequences in general, since this toy example also suffers from a selection bias – and the frequency itself of column patterns defines a phylogeny [17, 18]. Specifically, in this simulation experiment we generated very long multi-sequence data sets under UCA or IO (as in other simulations [2, 6]), reordered their columns based on their conservation (from higher to lower average identity), and then selected exhaustively subsets of columns along this reordered data sets such that the average identity was above a specific threshold. We used segments of 1000 columns, which were each subjected to the UCA test twice: once before and once after aligning the segments with MUSCLE [19].

**Fig. 2:**
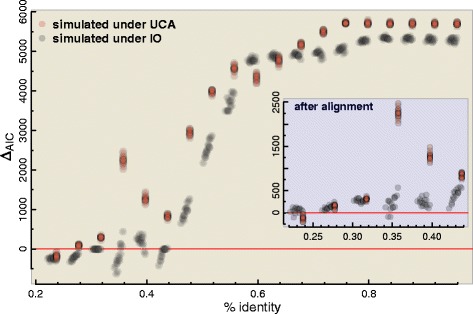
UCA test applied over large simulated data sets using a sliding window approach, where data sets’ columns were ordered from lower to higher average identity. Positive values for *Δ*AIC suggest a UCA. The inset shows AIC after optimizing the alignment within the segments (where the average identity refer to the segment before the alignment step)

The results are shown in Fig. 2, where we observe that the UCA hypothesis was always favoured whenever the average sequence identity threshold was higher than 0.44, even for sequences simulated under IO. Segments with similarities as low as 0.35 could also mislead the test in favour of UCA. And if we align the segments, then any sequences with more than 0.25 of average identity (before aligning) will be inferred as sharing a common ancestor, regardless of their actual relationship. Again, this is not an ideal simulation of highly similar IO sequences but still it suggests that by picking only the columns with high similarity we might falsely conclude for UCA. And importantly for our argument, it suggests that any reasonably conserved alignment would favour common ancestry under the UCA test no matter the actual origin of the sequences.

We could thus verify that the UCA test is oblivious to the source of the similarity: as long as the similarity is high enough it will favour UCA, while low similarities will have been previously camouflaged by an alignment optimization algorithm and even rejected altogether by BLAST or by the researcher, arbitrarily.

## A random permutation test

An interesting alternative to the UCA test that does not rely on high quality alignments is to apply a permutation test where the sites for some sequences are shuffled and then the AICs are recalculated after realignment, telling us how much the original data departs from those with phylogenetic structure partially removed. It is inspired by ([7] page 14), where it was suggested that the model selection test on one (or several?) shufflings would give an “upper bound for the effect of alignment bias”. This randomization test has similarities to the permutation tail probability (PTP) tests [20, 21], but would invalidate the AIC and Bayes factor (BF) interpretations since the support value for UCA cannot be interpreted in isolation^3^. If we must compare the AICs between the original and randomized replicates – all favouring UCA, as we have shown –, then we are back to a frequentist analysis, where e.g. the AIC alone represents just a summary statistic that cannot be interpreted as probabilistic support for one of the hypotheses. Furthermore, we must emphasize that the AIC comparison only has a probabilistic interpretation when evaluated under the same data – the alignment, unless explicitly accounted for by the model (for phyml [22], prottest [23] and others the data are the alignment columns). In other words, we cannot compare AICs between different alignments as suggested in [7], and even if we could, then this “discount” should be an intrinsic part of any formal test. However, although the original “upper bound” argument proposed by Theobald is mistaken, it can lead to a valid permutation test.

Indeed, many other statistics may work in such a frequentist approach, that don’t need to rely on AIC or LnL values. We therefore developed a randomization test where only simple summary statistics were considered, and applied it to *in silico* data sets. For each data set simulated under IO or UCA (same scenarios as in [2] Suppl. Mat.) we calculate the summary statistics and then we create a distribution of these statistics under the hypothesis of independent origins (H0), to which the original value is compared (the *p*-value). Each H0 replicate is created by changing the columns order for one of the groups in the original data set, as was done in ([1] section 3.1 of the suppl material) and described also in ([2] Suppl. Mat. section S2.2). Importantly, we always optimize the alignment for the original data set and each of the samples from H0 – so that we can estimate the ML tree, for instance. The summary statistics that we used were: 1) the sum of branch lengths of the ML tree for all sequences estimated under a LG model [24] using phyml; and 2) the average pairwise identity within groups minus the average pairwise identity between groups suspected of having independent ancestry. In both cases we expect lower values for UCA than for IO, and our *p*-value is thus constructed by counting the number of null-distributed replicates presenting a value as low as the original data (where “original data” is actually our multiple sequence alignments simulated with INDELible [13]).

**Fig. 3:**
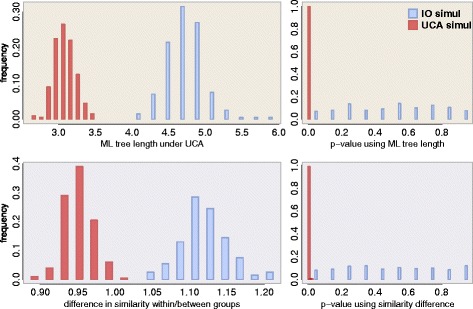
Frequentist non-parametric *p*-values where the null distribution was approximated by reshuffling columns of a subset of the sequences. On the left we have the distribution of the test statistics for the “original” sequences simulated under IO (*blue*) or under UCA (*red*), while the right show their associated *p*-values. At the top the test statistic is the maximum likelihood tree length under the common ancestry hypothesis, and at the bottom the statistic is the difference in average similarity within each group and between one group and the other

In Fig. 3 we show the results of 400 simulated data sets – 200 simulated under IO and 200 under UCA – where the null hypothesis was approximated by 100 shufflings (for each of the 400 data sets). We can see that not only the summary statistics are clearly different between IO and UCA data sets, but that the *p*-values can clearly distinguish both cases (with the *p*-value uniform under the null, as expected). We could have used the AIC or BF scores from the original UCA test as the comparison statistics here, but they are expected to give us similar results. Furthermore they would not give us any further insight, since their individual values would “support” UCA even under IO [2]. And as discussed below, even their differences or ratios would not represent statistical support any more because their comparison is illegitimate. Here we show again, as in [6], that the alignment properties are by themselves informative about UCA, and even without employing the whole AIC-based model selection analysis we can test for UCA. We should note that we do not endorse this test as the ultimate solution: as discussed by [21], the PTP test itself is flawed (but see [25]) and there might be caveats with our version as well. In any case, the “alignment bias” should not be used as a criterion for the adequacy of the UCA test, since the alignment step should be an integral part of any formal common ancestry test [2, 6].

### Aligning independently under each hypothesis

Model selection tests can help deciding between models for a given data set, but they cannot be compared across different data. Therefore we should not compare e.g. AIC values or log-likelihood ratios between different alignments, as under phyML and many other programs that do not consider explicitly the indel process. In contrast to e.g. [26], for these programs the data are the frequency of site patterns (i.e. the alignments). That is, alignment columns are considered independent and identically distributed observations from the evolutionary process. Therefore we stress that in order to apply the original UCA model selection test we must use the same alignment for both the IO and UCA hypotheses.

**Fig. 4:**
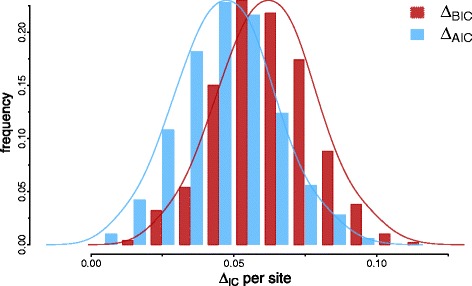
AIC and BIC per site if we align independently each subset (B and E sequences under IO, and B+E sequences under UCA). Each *Δ* *I* *C* (where IC = AIC or BIC) is calculated as *Δ* *I* *C*=*I* *C*(*B*)+*I* *C*(*E*)−*I* *C*(*B*+*E*) such that positive values favour UCA. The *Δ* *I* *C* values were further divided by the alignment length under UCA, to give scaled values comparable with other analyses

But what values would we observe if we could simply align the sequences independently? For this simulation we used the same simulation scenario as before [2] assuming an LG+IGF for each independently originated quartet – that we call B and E since they are based on bacterial and eukaryotic parameters, respectively. But now under the IO hypothesis we align the quartets separately – that is, in order to calculate the *AIC*(*B*) we align only the B sequences, and so forth. We can also try to account for the different alignment sizes by using the Bayesian Information Criterion (BIC) [27]:

$$BIC=k\log(N) - 2LnL $$

which is similar to the AIC but where we have the log of the number of data points (=column alignments in our case) instead of a fixed integer. The alignment size will be generally the same under each IO subset, and will correspond to the original sequence size of 6591 sites, while under UCA it will be around 10 *%* larger, indicating the imputation of indels if we align both subsets together [2]. The *ΔAIC* values do not change whether we align the putative independent data sets together or separately, and trying to correct for the alignment size makes the tests perform even worse (Fig. 4). Therefore, even if we align each subset independently from the others, we would still observe misleading, positive *ΔAIC*s. Again, the probabilistic interpretation of these information criteria is lost under this procedure.

## Discussion

In Theobald’s response to K&W’s simulations, he showed that by extending his test to include the true model (the MAX-Poisson under a star tree with infinite branch lengths, called “profile” model) it would be preferred over a single tree with a standard substitution model. This shows that the evaluated phylogenetic substitution models are consistent, but do not provide evidence about the appropriateness of the original UCA test. Even more, the actual model selection should be thought of as a blind test: we must not rely on some privileged knowledge about the true origin of the data set to reject hypotheses beforehand. Since we never know the true generating model of real data sets – which is especially true in phylogenetics – we must accept that all models we work with are misspecified [27].

On the other hand, if the inference for or against UCA depends on details of the phylogenetic model, then the test will only be useful when we know the true phylogenetic model. We do not expect a useful model to be very sensitive to model violations, especially when these violations can be assumed to affect both hypotheses. We expect the test to favour the correct hypothesis for any model close enough to what might be the true generating one.

For example if our conclusion for UCA or IO changes depending on whether allow or not rate heterogeneity, whether we include or not a given replacement matrix, or some other mild model misspecification, then it becomes hard to defend our conclusion, and we should not trust this model selection. Our expectation is that a model good enough will affect both hypotheses likewise.

We are not against extending the UCA test framework to include more models, which might help distinguishing an IO data set from an UCA one. After all, the test output will give the odds ratio given a set of assumptions – like for instance rate heterogeneity, common branch lengths along the alignment, a common topology for all sites, etc. And we can always improve on the assumptions. Furthermore if we can devise an evolutionary model whereby independent sequences can mislead BLAST searches and alignment procedures, certainly we would like to see it implemented it in such a model selection framework. But we should accredit it as a contribution to a better model selection test, particularly if such model could have systematically misled the original one. Systematically misleading simulations are a valid criticism to a particular model selection scheme, that deserve credit.

We should not dismiss a model based solely on our subjective impressions about commonplace data sets, either: novel methodologies are created precisely to discover patterns that were hidden or unexplained so far. Therefore biological realism or representativeness may not be good judges of a model’s relevance. In exploratory analysis we employ several short cuts like skipping similar models or disregarding those based on assumptions known to be very unlikely. But when the aim is to assign objectively probabilities to the hypotheses, then we should consider and embrace models capable of refuting them.

A more serious problem may be when model misspecification happens only under one of the hypothesis (due to software limitations, for instance). For instance, cases where amino acid replacement model heterogeneity between the independently evolved data sets can affect the test: while under UCA all branches are forced to follow the same replacement matrix, gamma parameter and equilibrium frequencies, under IO the independently evolved groups are allowed to have their own ones. We recognize that this is an implementation problem and not a theoretical one – programs usually make this homogeneity assumption to avoid overparameterization. Nonetheless, we should be careful whenever the test favours IO since it might be the case of a better parameterization – one set of parameters for each subtree. Whenever the test favours IO, we should always try to isolate the effect of the IO assumption against the confounding effect of amino acid replacement heterogeneity by one of two ways.

One is by extending the software to replace the fixed parameter by a variable one. That is, to allow the implemented model to have a variable replacement matrix along the tree, or a heterogeneous equilibrium frequency vector across branches, etc. so as the UCA tree can access the same parameter space as the IO trees. The other is to assume homogeneity under the IO hypothesis by using the same parameters over all independently evolved groups, such that any model misspecification can be “marginalized”. If some apparent support for the IO hypothesis disappears once we force homogeneity, then we can suspect that the model misspecification was misleading the test.

We maintain that the UCA test as originally proposed [1] is heavily biased towards UCA, but a good counterargument would be to show a replicable simulation procedure that generates bias-free alignments where the test correctly detects IO. The problem lies in that there are no known mechanisms (at least none that we are aware of) by which we can simulate independently evolved sequences that satisfy the quality requirements imposed in [7] – and any attempt might be met with a special pleading, as we have seen. It is worth noticing that another method has been recently proposed that can more directly test for ancestral convergence [12]. This method does not seem to suffer from the drawbacks of the UCA test, since it takes into account the alignment step.

Another powerful argument for the common ancestry of life is to show how distinct genes or different units of information support similar phylogenetic histories – and we can only thank Douglas Theobald for the herculean task of compiling the evidence for it in an accessible manner (http://www.talkorigins.org/faqs/comdesc/). But unfortunately the opportunity of showing this consilience of trees for the universally conserved proteins was missed: the UCA model selection framework suggested that several trees were much more likely than a single tree for all proteins [1], which *prima facie* goes against a universal phylogeny, in the absence of a quantification of the amount of disagreement. We are thus left only with a visual corroboration of the non-random clustering of taxa ([1] Figure 2a), which do indeed provide evidence for the common ancestry of the analysed sequences.

## Conclusions

We have shown that the K&W profile model [3] was a valid simulation of sequences with independent origins where the UCA test described in [1] indeed fails. We have also shown that the UCA test does not correctly infer the independent origins of sequences simulated under a simpler profile model, even when the model is among those being tested. We then proceeded to show that even if we restrict ourselves to sequences that look similar, the UCA test as proposed in [1] would still be biased towards UCA. Finally, after discussing the inappropriateness of comparing likelihoods between different data sets — as has been suggested in [7], for instance — we devise frequentist permutation tests that do not seem to have the drawbacks of the original UCA test.

In summary, we conclude that for many data sets the original UCA test cannot reject the UCA hypothesis even in the absence of a common ancestor, where this failure can only be downplayed by subjectively excluding the problematic data sets.

## Reviewers’ comments

### Reviewer’s report 1: Prof Eugene Koonin

#### Reviewer summary

This is a carefully performed, technically sound re-examination of the UCA test published by Theobald. De Oliveira Martins and Posada treat an alignment of unrelated sequences (the IO case) as a star tree with arbitrary branch lengths, and using this approach, validate the previous conclusion of Koonin and Wolf that the UCA test in effect relies on sequence similarity. Given that similarity is high enough, the test is heavily biased towards the UCA hypothesis and fails to provide an objective refutation of the IO hypothesis. I do not see any substantial problems with the analysis. The paper is quite technical in character and as such, can be fully appreciated only by practicing phylogeneticists. However, given the fundamental importance of the UCA problem, the conclusions at least will be of interest to many biologists.

#### Reviewer recommendations to authors

I see no major problems with the manuscript.

#### Minor issues

The English usage merits some attention. For example, the authors systematically use ‘specially’ instead of ‘especially’, this has to be fixed. The use of apostrophes (didn’t, shouldn’t) is not advisable. The work of Theobald published in 2010 hardly can be considered ‘recent’ let alone ‘very recent’ as claimed in the abstract. There are some typos as well.

Authors’ response: *We changed the text, following the suggestions, and also fixed all typos.*

### Reviewer’s report 2: Dr Yuri Wolf

#### Reviewer summary

The manuscript by de Oliveira Martins and Posada explore the ability of phylogenetic tests to distinguish between common ancestry and independent origin of sequences. The paper touches upon deep issues of phylogenetic analysis and is of considerable interest.

#### Reviewer recommendations to authors

de Oliveira Martins and Posada further explore the controversy with the so called Universal Common Ancestry (UCA) test, introduced by Theobald in his 2010 paper [[1] in the current manuscript]. The authors validate the original criticism by Koonin and Wolf [3], demonstrating that the original UCA test is unable to discriminate between the meaningful phylogeny and a star tree with infinitely long branches (equivalent to an independent origin). de Oliveira Martins and Posada show that the UCA test doesn’t perform even when a variety of modifications that might have taken care of several shortcomings of the test (overspecification of the profile model, alignment bias etc). They conclude that any plausible test is heavily biased towards supporting common ancestry even when the sequences are explicitly not related phylogenetically. In my opinion the most interesting part of the current work is the more general conclusions that all existing methods of phylogenetic analysis seem to be strongly predicated on the a priori existence of meaningful phylogeny and are unable to reject this hypothesis almost by design. This suggests that development of the analytical framework that is capable of tackling such questions might require going outside of the “classical” phylogenetics paradigm.

Authors’ response: *We believe that this circularity is due to neglecting the effects of the alignment, since each column in a fixed aligment is a statement of homology. Therefore phylogenetic models that can circumvent this limitation at least in theory could test more objectively for the appropriateness of finite branch lengths.*

### Reviewer’s report 3: Prof William Martin

#### Reviewer summary and recommendations to authors

This is a continuation of the discourse precipitated by the Theobald paper in Nature a few years ago. Martins and Posada weigh in with some very incisive insights regarding models to test the predictions of universal common ancestry as formulated by Theobald. I also reviewed Koonin’s response to Theobald in these pages a few years back, it was good, this is even better. I think this paper should be published, it is a valuable contribution to the debate. It is an even more valuable contribution to advances in the realm of models, which are getting very complex anymore. I would like to see Posada, a leading expert on models, someday address the issue that the most highly parameterized models seem to always get the best likelihoods, and how that figures in to understanding deep phylogeny, but that is a different paper for a different day. I am worried that by adding too many parameters to models we are getting the best likelihoods, but not at the expense of listening more to what the models say than to what the data say. That also comes to the fore in this paper. After all, with today’s large data sets with many sites per OTU, what we seem to be getting are fully resolved trees that differ across models, such that the models are slowly becoming more important than the data in phylogeny, an interesting development. At any rate, I like this paper a lot, it can be published as is (I found one typo: “Inthis”)

Authors’ response: *For nested models, adding parameters can only result in equal or better likelihods. For real data we normally see an increase. However, we have criteria like AIC or BIC to penalize overfitting. In fact, the more data we have (and this is the trend for phylogenomics), the likelihood, P(D∣M), will become more and more important compared to the number of parameters. Rather than the number of parameters, in our opinion the key, given a large amount of data, is which parameters do we include. Only by adding meaningful parameters (say variable frequencies across the alignment) we will be able to listen more from the data.*

## Endnotes

^1^ equals one if and only if *χ* is true and equals zero otherwise

^2^ Although we must never expect real data sets to follow the implemented model exactly

^3^ We cannot compare likelihoods between different data

## Additional file

Additional file 1
**Appendix**. Derivation of the independent origins model as a special case of the common origin where some branch lengths are fixed. (PDF 108 kb)

## Abbreviations

UCA: universal common ancestry
CA: common ancestry
IO: independent origins
K&W: Koonin and Wolf [3]
AIC: akaike information criterion
PTP: permutation tail probability
BF: Bayes factor
