# Supplementary material for: Infinitely long branches and an informal test of common ancestry
Source: Biol Direct. 2016 Apr 7;11:19. doi: 10.1186/s13062-016-0120-y (PMC4823899; doi:10.1186/s13062-016-0120-y)
Supplement: Additional file 1 — Appendix. Derivation of the independent origins model as a special case of the common origin where some branch lengths are fixed. (PDF 108 kb) [file 13062_2016_120_MOESM1_ESM.pdf]

Infinitely Long Branches and an Informal Test of Common Ancestry

Leonardo de Oliveira Martins*[1,2] and David Posada†[1]

[1] *Department of Biochemistry, Genetics and Immunology, University of Vigo, Spain*

[2] *Department of Materials, Imperial College London, UK*

APPENDIX – Independent origins as a special case of the common origin

Here we show in more detail our sketched proof that in the model selection proposed by [1] the IO hypothesis is a particular case of the model for UCA, if we assume a single evolutionary model M [2, Suppl Mat]. However, this conclusion remains valid if we relax the fixed model assumption: instead of a single evolutionary model we can think of variable models along the tree.

The following diagram represents how the UCA model (at the left) can lead to the IO model (at the right), where we see that after the "removal" of the internal branch the remaining neighboring branches have one less degree of freedom since the likelihood is the same whenever their sum is the same. In other words, for each independent origin three internal branches are fixed: one at ∞, representing the *de novo* appearance, and one at each of its sides becoming redundant by the pulley principle. The parameters a and c are constants between zero and one and a natural choice is $a = c = 1$, while A, B, C and D are subtrees (with one or more leaves).

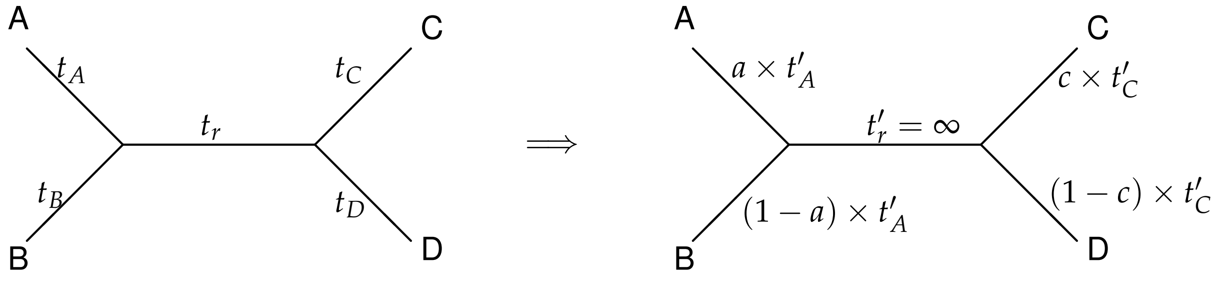

*leomrtns@gmail.com

†dposada@uvigo.es

The justification for fixing the branch length at infinity comes from the fact that the Markov chains used in amino acid replacement models converge to their equilibrium distributions. That is, the probability $P(x \mid z, t, M)$ of going from state z to state x in time t under evolutionary model M becomes independent of z when $t \to \infty$, and approaches the equilibrium frequency π_x of x under model M:

$$\lim_{t\to\infty} P(x \mid z, t, M) = \pi_x$$

The likelihood $P(X \mid T, \mathbf{t}, M)$ of a phylogenetic tree T with branch length vector $\mathbf{t}$ arbitrarily rooted at r can be calculated for a given alignment column X as

$$P(X \mid T, \mathbf{t}, M) = \sum_z \pi_z L_r(z \mid \mathbf{t}, M) \tag{1}$$

where $L_r(z \mid \mathbf{t}, M)$ is the partial likelihood of node r for amino acid state z, and can be calculated recursively by

$$L_r(z \mid \mathbf{t}, M) = \Big[\sum_x P(x \mid z, t_a, M) L_A(x \mid \mathbf{t}, M)\Big]\Big[\sum_y P(y \mid z, t_b, M) L_B(y \mid \mathbf{t}, M)\Big] \tag{2}$$

Assuming the subtree with branch lengths $t_a, t_b \in \mathbf{t}$ connecting r to (internal or external) nodes A and B represented by

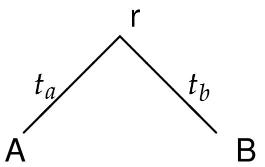

In the case when t_a and t_b go to infinity then as we saw $P(x \mid z, t_a, M) = \pi_x$ and $P(y \mid z, t_b, M) = \pi_y$, and therefore we have that equation 2 reduces to

$$L_r(z \mid \mathbf{t}, M) = \Big[\sum_x \pi_x L_A(x \mid \mathbf{t}, M)\Big]\Big[\sum_y \pi_y L_B(y \mid \mathbf{t}, M)\Big] = W \tag{3}$$

which is independent of z, and thus the site likelihood of equation 1 becomes

$$P(X \mid T, \mathbf{t}, M) = \sum_z \pi_z L_r(z \mid \mathbf{t}, M) = \sum_z \pi_z W = W \sum_z \pi_z = W \tag{4}$$

By comparing each of the two terms in equation 3 and equation 1 we can see that W is the

product of the site likelihoods of two independent trees under the model described in [1], arbitrarily rooted at A and B. That is, if we represent the likelihood under the IO hypothesis as calculating independently the likelihoods of the subtrees rooted at A and B and multiplying them, then we have that each of these terms will be $\sum_x \pi_x L_\rho(x \mid \mathbf{t}, M)$ where $\rho = (A, B)$, as in equation 1. The product of these terms is identical to the likelihood of a single tree with an infinite branch length connecting A and B, described by equation 4.

The extension for distinct amino acid replacement models over the tree is straightforward (replacing M by $\mathbf{M} = (M_1, \ldots, M_{2N-2})$ for N leaves), with the caveat that despite it can be handled by sequence simulation programs like INDELible [3], it is not implemented yet in popular phylogenetic reconstruction methods. Therefore the lack of correspondence of models M between the hypotheses is a limitation of the software employed, and not of the hypothesis test as devised.

References

[1] Douglas L Theobald. A formal test of the theory of universal common ancestry. *Nature*, 465(7295):219–22, May 2010.

[2] L. de Oliveira Martins and D. Posada. Testing for universal common ancestry. *Systematic Biology*, 63(5):838–842, Jun 2014.

[3] William Fletcher and Ziheng Yang. INDELible: a flexible simulator of biological sequence evolution. *Molecular biology and evolution*, 26(8):1879–88, August 2009.
